# Supplementary material for: Whole Genome Analysis and Assessment of the Metabolic Potential of Streptomyces carpaticus SCPM-O-B-9993, a Promising Phytostimulant and Antiviral Agent
Source: Biology (Basel). 2024 May 28;13(6):388. doi: 10.3390/biology13060388 (PMC11200584; doi:10.3390/biology13060388)
Supplement: Supplementary file 1 [file biology-13-00388-s001.zip › biology-2972496-supplementary.pdf]

Fig. S1. Emergence of colonies of *S. carpaticus* strain SCPM-O-B-9993 on potato agar on cultivation day 14

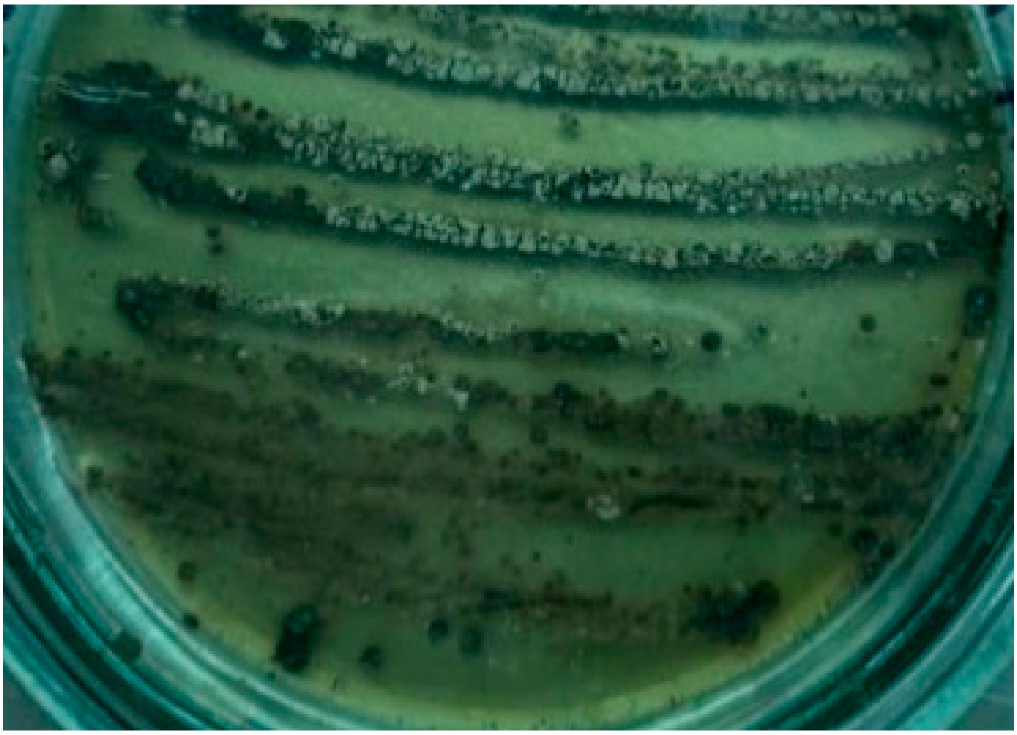

Table S1. Phytotoxicity of *S. carpaticus* strain SCPM-O-B-9993

Incubation time, hrs	Root length, mm	Stem length, mm	Germination, average value, pcs. (%)	Biomass, g
24	3.74±1.22	3.66±1.24	17.0 (56.7)	0.125±0.03
48	2.55±1.55	3.97±2.43	14.0 (46.7)	0.127±0.03
72	23.89±18.02	13.31±10.44	18.5 (61.7)	0.205±0.11
96	7.00±2.43	4.38±1.54	8.5 (28.3)	0.049±0.08
120	10.67±2.54	8.21±3.43	18.0 (60.0)	0.273±0.15
144	4.13±1.78	8.23±7.29	19.3 (64.3)	0.239±0.07
168	2.81±0.75	5.06±1.06	8.5 (28.3)	0.022±0.01
K+	10.63±8.09	12.47±10.03	9.0 (30.0)	0.240±0.14
K-	7.09±4.46	6.13±3.88	11.0 (36.7)	0.205±0.11

Fig. S2. Manifestation of symptoms of the cucumber mosaic virus on day 7 of exposure after the first treatment: (A) control; (B) the experiment (exposure to the strain)

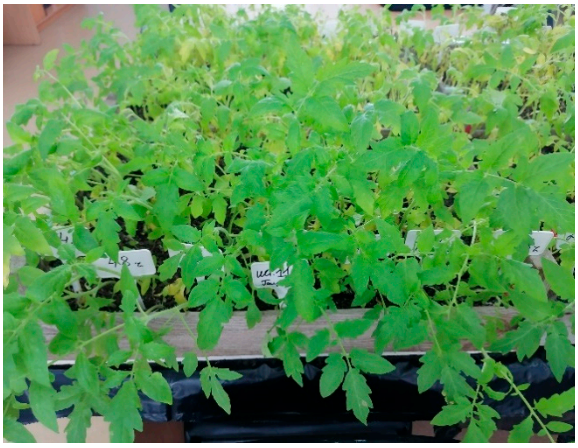

A

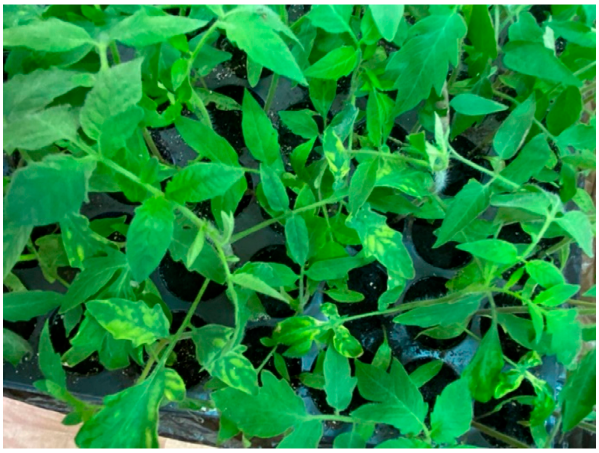

B

Table S2. Search for strains most related to strain SCPM-O-B-9993 using marker genes (housekeeping genes). The same strains are highlighted with one color

*gyr*B

	Query cover, %	Percent identity, %	Genome length, Mb	Genbank accession number
Streptomyces harbinensis strain NA02264	100%	99.70%	5.80	CP054938.1
Streptomyces sp. XC 2026 chromosome	99%	91.71%	5.83	CP064057.1
Streptomyces xiamenensis strain 318	100%	91.43%	5.96	CP009922.3

*rpo*B

Streptomyces harbinensis strain NA02264	100%	99.83%	5.80	CP054938.1
Streptomyces sp. XC 2026 chromosome	100%	96.39%	5.83	CP064057.1
Streptomyces xiamenensis strain 318	100%	96.33%	5.96	CP009922.3
Streptomyces marincola strain SCSIO 64649	100%	92.17%	6.62	CP084541.1
Streptomyces marincola strain SCSIO 03032	100%	91.91%	6.28	CP021121.1
Streptomyces sp. MA3_2.13	100%	91.17%	7.65	CP082362.1
Streptomyces radiopugnans strain MRC003	100%	91.14%	6.29	CP094495.1

*rec*A

Streptomyces harbinensis strain NA02264	100%	99.39%	5.80	CP054938.1
Streptomyces xiamenensis strain 318	100%	93.41%	5.96	CP009922.3
Streptomyces sp. XC 2026	100%	92.99%	5.83	CP064057.1

*trp*B

Streptomyces harbinensis strain NA02264	100%	99.60%	5.80	CP054938.1
Streptomyces xiamenensis strain 318	98%	93.54%	5.96	CP009922.3
Streptomyces sp. XC 2026	98%	93.04%	5.83	CP064057.1
Streptomyces marincola strain SCSIO 64649	97%	90.02%	6.62	CP084541.1

Table S3. Distribution of coding sequences unique to the SCPM-O-B-9993/NA02264 pair by biological processes

GO:0005975	carbohydrate metabolic process	5
GO:0005976	polysaccharide metabolic process	2
GO:0006066	alcohol metabolic process	2
GO:0006081	cellular aldehyde metabolic process	1
GO:0006082	organic acid metabolic process	9
GO:0006091	generation of precursor metabolites and energy	1
GO:0006139	nucleobase-containing compound metabolic process	19
GO:0006281	DNA repair	1
GO:0006464	cellular protein modification process	1
GO:0006508	proteolysis	3
GO:0006518	peptide metabolic process	3
GO:0006629	lipid metabolic process	6
GO:0006725	cellular aromatic compound metabolic process	25
GO:0006766	vitamin metabolic process	3
GO:0006793	phosphorus metabolic process	3
GO:0006805	xenobiotic metabolic process	2
GO:0006807	nitrogen compound metabolic process	31
GO:0006810	transport	12
GO:0006811	ion transport	6
GO:0006865	amino acid transport	2
GO:0007154	cell communication	1
GO:0008150	biological process	57
GO:0008152	metabolic process	61
GO:0009116	nucleoside metabolic process	1
GO:0009117	nucleotide metabolic process	3
GO:0009225	nucleotide-sugar metabolic process	1
GO:0009308	amine metabolic process	1
GO:0009987	cellular process	33
GO:0015031	protein transport	1
GO:0015833	peptide transport	2

GO:0016043	cellular component organization	1
GO:0016070	RNA metabolic process	15
GO:0017144	drug metabolic process	4
GO:0019538	protein metabolic process	6
GO:0019748	secondary metabolic process	5
GO:0032502	developmental process	2
GO:0032989	cellular component morphogenesis	1
GO:0042180	cellular ketone metabolic process	1
GO:0042440	pigment metabolic process	3
GO:0042445	hormone metabolic process	2
GO:0043170	macromolecule metabolic process	23
GO:0043603	cellular amide metabolic process	4
GO:0044237	cellular metabolic process	50
GO:0044238	primary metabolic process	27
GO:0044255	cellular lipid metabolic process	4
GO:0044419	interspecies interaction between organisms	1
GO:0045333	cellular respiration	1
GO:0046483	heterocycle metabolic process	25
GO:0050896	response to stimulus	12
GO:0051186	cofactor metabolic process	10
GO:0051234	establishment of localization	12
GO:0051604	protein maturation	1
GO:0051704	multi-organism process	2
GO:0065007	biological regulation	13

Fig. S3. Schemes of genetic organization of highly conserved clusters of secondary metabolite biosynthesis: (a) ohmyungsamycin A/ohmyungsamycin B, (b) pellasoren, (c) ectoine, (d) coelibactin, (e) naringenin, (f) ε-Poly-L-lysine

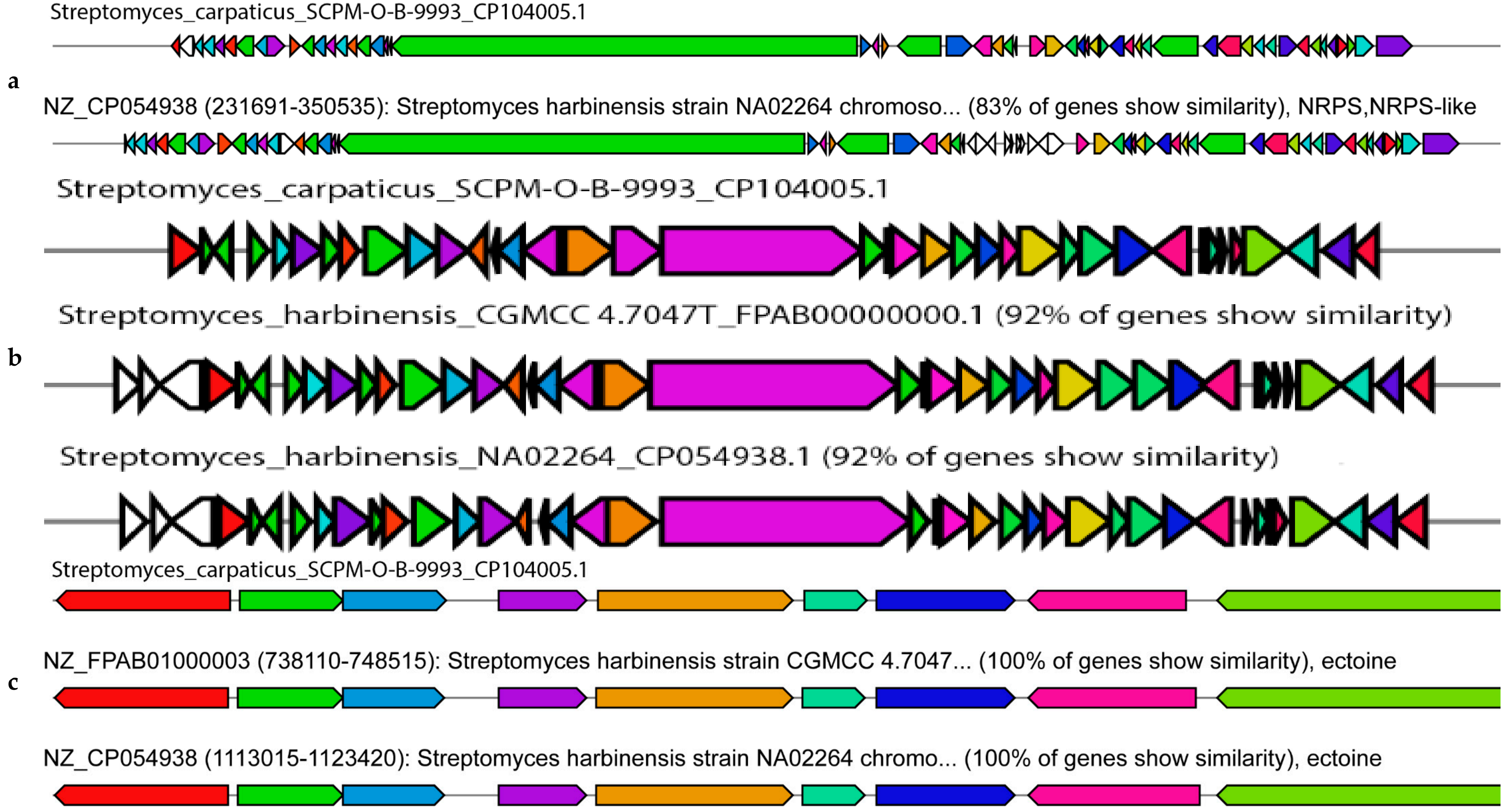

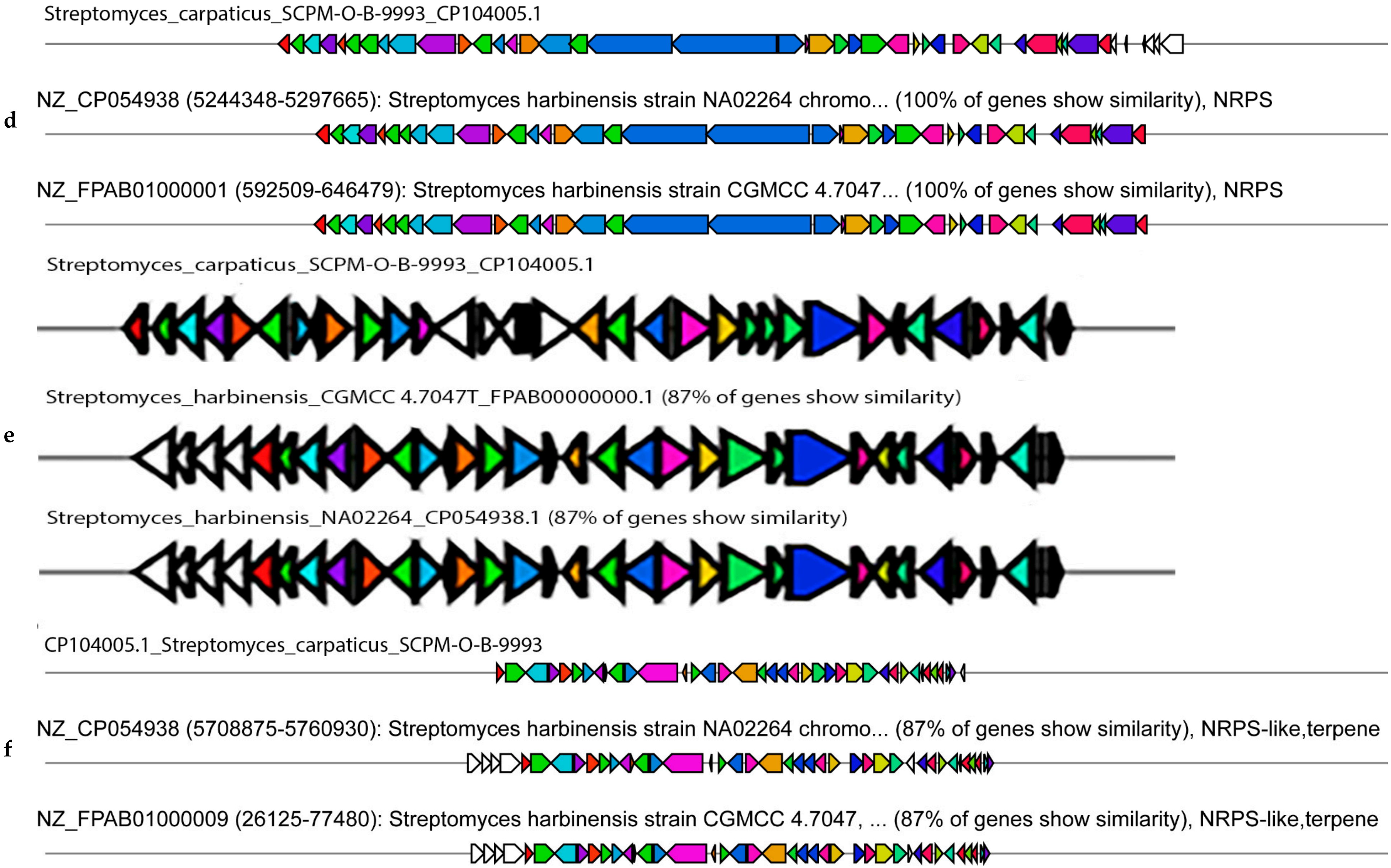
d
Streptomyces_carpaticus_SCPM-O-B-9993_CP104005.1
NZ_CP054938 (5244348-5297665): Streptomyces harbinensis strain NA02264 chromo... (100% of genes show similarity), NRPS
NZ_FPAB01000001 (592509-646479): Streptomyces harbinensis strain CGMCC 4.7047... (100% of genes show similarity), NRPS
e
Streptomyces_carpaticus_SCPM-O-B-9993_CP104005.1
Streptomyces_harbinensis_CGMCC 4.7047T_FPAB00000000.1 (87% of genes show similarity)
Streptomyces_harbinensis_NA02264_CP054938.1 (87% of genes show similarity)
f
CP104005.1_Streptomyces_carpaticus_SCPM-O-B-9993
NZ_CP054938 (5708875-5760930): Streptomyces harbinensis strain NA02264 chromo... (87% of genes show similarity), NRPS-like,terpene
NZ_FPAB01000009 (26125-77480): Streptomyces harbinensis strain CGMCC 4.7047, ... (87% of genes show similarity), NRPS-like,terpene

Table S4. Regions in conserved clusters of secondary metabolite biosynthesis containing extended mismatched regions

Pair **SCPM-O-B-9993/NA02264**	Pair **SCPM-O-B-9993/CGMCC4.7047T**	Gene product
**coelibactin**		
5398212 ggtgggttcggacggtccggaaggagcgggggaggcggacgggggacaggggacgggcag 5398271<br>5251488 GGTGGAT--GG--GTT-CGGATGGTCCGGA--AGGA--ACGGGGG------------AG 5251525	5398201 TTCCCTgggtgggtgggttcggacggtccggaaggagcgggggaggcggacgggggacag 5398260<br>639309 TTCCCTGGGTGGGTGG-AT--G--GGTTCGGATGG-TC--------CGGA---AGGA-A- 639269<br><br>5398261 gggacgggcagggggcccgggggcGAGATCAGCCGAGGCAGGCGTACAGCGAGTCCGCGT 5398320<br>639268 CGG--GGG-AGGGGGCCCGGGGGCGAGGTCAGCCGAGGCAGGCGTACAGCGAGTCCGCGT 639212	intergenic region
5409843 GCGGCCGAAGTTGGTGCCCGCGACCACGGTGCGCAGcggacggccgggggacggcgggga 5409902<br>5262788 GCGGCCGAAGTTGGTGCCCGCGACCACGGTGCGCAGCGGACGGCCGGGGGACGG------ 5262841<br><br>5409903 cgccggggacggcgggacggTCACAGGGCACCGTCCTCGAACGCGAGCTGCGCCGGGGCG 5409962<br>5262842 ---CGGGGACGGCGGGACGGTCACAGGGCACCGTCCTCGAACGCGAGCTGCGCCGGGGCG 5262898	5409901 gacgccggggacggcgggacggTCACAGGGCACCGTCCTCGAACGCGAGCTGCGCCGGGG 5409960<br>627631 GACG---------GCGGGACGGTCACAGGGCACCGTCCTCGAACGCGAGCTGCGCCGGGG 627581	Gfo/Idh/MocA family oxidoreductase
	5417158 GTGCGGTACCAGCGCCCGGTGCCGTCGCCGGACGGGGCGGACGGG---------TCACCG 5417208<br>620380 GTGCGGTACCAGCGCCCGGTGCCGTCGCCGGACGGGGCGGACGGGGCGGACGGGTCACCG 620321	amino acid adenylation domain-containing protein
5429522 ggccggccacgccggtaccgccgtccgcgccgcctgccccgtccg-c----g-ccgtccg 5429575<br>5282459 GGCCGGCCGCGCTGGTACTGCCGTCCGCGCCGCCCGGGCCGTCCGGCTCCCGGCCGCCCG 5282518<br><br>5429576 -gaa-------------------------cccggccgcccgtGCTGGAACTGACGGGGC 5429608<br>5282519 CGCCGCCCGCGCCGCCCGGGCCGTCCGGCTCCCGGCCGCCCGTGCTGGAACTGACGGGGC 5282578	5429567 c----g-ccgtccg-gaa-cccg-gccgcccgtGC--T--GGAACT-GAC-G---G-G-- 5429606<br>607960 CTCCCGGCCGCCCGCGCCGCCCGCGCCGCCCGGGCCGTCCGGCTCCCGGCCGCCCGTGCT 607901<br><br>5429607 G-----------CTGCGGGCCGGCCATGGGCGGCGGATCACCGTGGCGGGGGTGTCCCT 5429654<br>607900 GGAACTGACGGGACTGCGGGCCGGCCACGGGCGGCGGATCACCGTGGCGGGGGTGTCCCT 607841	ATP-binding cassette domain-containing protein
5432489 CCGGTGCGC-CC-C--GT--TCCCCGTTCTC--T-----GTTCTCTGTTCCCC--TTTCC 5432533<br>5285456 CCGGTGCGCTCCGCCCGCGCTCCCCGTTCCCCGTTCCCCGTTCTCTGTTCCCCTTTTTCC 5285515	5432475 CGGGCCTGCGCCGTCCGGTGCGC-CC-C--GT--TCCCCGTTCTC--T-----GTTCTCT 5432521<br>605020 CGGCCCGGCGCCGTCCGGTGCGCTCCGCCCGCGCTCCCCGTTCCCCGTTCCCCGTTCTCT 604961	intergenic region
	5437859 gccccgggccgccggggctcggcggtgg-----------t-gg---g--g---ggcggg 5437897<br>599628 GCCCCGGGCCGCCGGGGCTCGGCGGTGGCGGGCGGCGGGGTCGGCCCGACGGGCGGCGGG 599569	intergenic region
5438101 AGTCGGGGGAGCCGGGGGAGCCGGCCACGGGGCTCGGTCAGCTGACgcggtgggtggcgt 5438160<br>5291094 AGTCGGGGGAGT---------CGGCCACGGGGCTCGGTCAGGTGACGCGGTGGGTGGCGT 5291144	5440706 ccggccgccgccccggcgcc--------------------ggccgtgaccgagccgcTG 5440744<br>596766 CCGGCCGCCGCCCCGGCGCCCGTACCGCCCGCCCCGGCGCCGGCCGTGACCGAGCCGCTG 596707	intergenic region
5440660 GGACAGCCCGATGTACAGGCAGTccccgggggccggcgcggtgccgccggccg------- 5440712<br>5293608 GGACAGCCCGATGTACAGGCAGTCCCCGGGGGCCGGCGCGGTGCCGCCGGCCGCCGCCCC 5293667<br><br>5440713 --------------ccgccccggcgccggccgtgaccgagccgcTGAAGCACGGCACGTC 5440758<br>5293668 GGCGCCCGTACCGCCCGCCCCGGCGCCGGCCGTGACCGAGCCGCTGAAGCACGGCACGTC 5293727		putative baseplate assembly protein
**pellasoren**		

<table>
<tr><td>560305 GGATCAGACGATCCGGCAGCCgctgcgggcgacccgcggcggcgagtgggtgcggctgcg 560364
||| ||||||||||||||||||||||||||||||||||||||||||||||||
456090 GGACCAGACGATCCGGCAGCCGCTGCGGGCGACCCGCGGCGGCGAGTGGG---------- 456139

560365 gctgcggctgcggctgcggctgTCCAACGTGTACGGCACCGGGCCGCTCACCGTGGACGC 560424
|||||||||||||||||| ||||||||||||||||||||||||||||||
456140 --------TGCGGCTGCGGCTGTCCAATGTGTACGGCACCGGGCCGCTCACCGTGGACGC 456191</td><td>560368 gcggctgcggctgcggctgTCCAACGTGTACGGCACCGGGCCGCTCACCGTGGACGCGCT 560427
| |||||||||||||||||||||||||||||| |||||||||
612094 G------------------TCCAACGTGTACGGCACCGGGCCGCTCACCGCGGACGCGCT 612053</td><td>hypothetical protein</td></tr>
<tr><td colspan="2">naringenin</td><td></td></tr>
<tr><td>5836406 cgcaccggacggctccccgtccgcct-c--cggccgtc--cgtccccctccggcggcccg 5836460
|||||||| |||||||||| ||||| | |||| ||| ||||| |||||||||||||
5678012 CGCACCGGCCGGCTCCCCG-CCGCCCGCGTCGGC-GTCGGCGTCCGGCTCCGGCGGCCCG 5678069</td><td>5836406 cgcaccggacggctccccgtccgcct-c--cggccgtc--cgtccccctccggcggcccg 5836460
|||||||| |||||||||| ||||| | |||| ||| ||||| |||||||||||||
108340 CGCACCGGCCGGCTCCCCG-CCGCCCGCGTCGGC-GTCGGCGTCCGGCTCCGGCGGCCCG 108283</td><td>biotin synthase BioB</td></tr>
<tr><td>5838860 gccgctcttccctcctccgccgtccttgctgccgcccttgccgtccttcccgcctccgcc 5838919
|||||||||||| |||||||||||||| | ||||| | ||| || ||| |||||
5680470 GCCGCTCTTCCCGCCTCCGCCGTCCTT-C--CCGCC-T--CCG-CCGTCC----TCCGC- 5680517</td><td>5838861 ccgctcttccctcctccgccgtccttgctgccgcccttgccgtccttcccgcctccgccg 5838920
||||||||||| ||||||||||||| | ||||| | ||| || ||| ||||| |
105882 CCGCTCTTCCCGCCTCCGCCGTCCTT-C--CCGCC-T--CCG-CCGTCC----TCCGC-G 105835</td><td>non-homologous end-joining DNA ligase</td></tr>
<tr><td>5872889 ccttccccgccgccccgcgccgccccgccccgcgccgctcacgccccggcgttcgccgcc 5872948
|||||||||||||| ||| || | ||| |||||||||||||||| ||||||||
5708618 CCTTCCCCGCCGCCTCGC-CC-C---GCC-----CCGCTCACGCCCCGGCGCTCGCCGCC 5708667</td><td></td><td>intergenic region</td></tr>
<tr><td colspan="2">ohmyungsamycin</td><td></td></tr>
<tr><td>364934 TCAGTCCTCTCggtgggtggagtggatgtggtgggtgtggtggCTGCGGAGTTCCGCGGC 364993
|||||||||||||||||||| |||||||||||||||||||||||||||||||
250768 TCAGTCCTCTCGGTGGGTGG---------GGTGGGTGTGGTGGCTGCGGAGTTCCGCGGC 250818</td><td></td><td>non-ribosomal peptide synthase/polyketide synthase</td></tr>
<tr><td>404294 CATCTCCGGCAG---------CTCC-G--GCGTCTGGGGGGCGGCAGGCGCGCCGCCGCG 404341
|||||||||||| |||| | ||||||| ||| |||| ||||||||||||||
290116 CATCTCCGGCAGTACCGGCAGCTCCGGCCGCGTCTGCGGGTCGGCGGGCGCGCCGCCGCG 290175</td><td></td><td>non-ribosomal peptide synthase/polyketide synthase</td></tr>
<tr><td>409553 TTCTGCGGGCCGTCCGAACCGCCGCTCTCGCCCCCGGTGTTGCCCCCGGCGTCAccgccg 409612
||||||||||||||||||||||||||||||||||||||||| || | || |||
295392 TTCTGCGGGCCGTCCGAACCGCCGCTCTCGCCCCCGGTGT---------CG-C-CC-CCG 295439</td><td></td><td>non-ribosomal peptide synthase/polyketide synthase</td></tr>
<tr><td colspan="2">ε-poly-L-lysine</td><td></td></tr>
<tr><td>5879455 CGCGCT------------CACCGCACCCCGGTACGCCCTCGTCCTGGCCCTCGCCCTCGC 5879502
|||||| ||||||||||||||||||||||||||||||||||||||||||
5715183 CGCGCTCACCGCCCGGCGCACCGCACCCCGGTACGCCCTCGTCCTGGCCCTCGCCCTCGC 5715242</td><td>5879455 CGCGCTCACCGC------------ACCCCGGTACGCCCTCGTCCTGGCCCTCGCCCTCGC 5879502
|||||||||||| ||||||||||||||||||||||||||||||||||||
71172 CGCGCTCACCGCCCGGCGCACCGCACCCCGGTACGCCCTCGTCCTGGCCCTCGCCCTCGC 71113</td><td>SGNH/GDSL hydrolase family protein</td></tr>
<tr><td>5887963 ccgggccccgcgcgccccgcgacccccgcgcaccacccgccgcccgcgccgcgcccgtgc 5888022
||||||||||||| |||||||||||||||||||||||||||||||||||||||
5723702 CCGGGCCCCGCGC---------CCCCCGCGCACCACCCGCCGCCCGCGCCGCGCCCGTGC 5723752</td><td>5887963 ccgggccccgcgcgccccgcgacccccgcgcaccacccgccgcccgcgccgcgcccgtgc 5888022
||||||||||||| |||||||||||||||||||||||||||||||||||||||
62653 CCGGGCCCCGCGC---------CCCCCGCGCACCACCCGCCGCCCGCGCCGCGCCCGTGC 62603</td><td>carbohydrate ABC transporter permease</td></tr>
<tr><td>5897380 cccggcgcgcgggcgtgccccggcgcacggccggaccggcttggcggcagcgggcggggg 5897439
| | | |||||||||||||||||||||||||||||| || |||||||||||||||
5733106 ---G-C-C---GGCGTGCCCCGGCGCACGGCCGGACCGGCGCGGTGGCAGCGGGCGGGGG 5733157</td><td></td><td>intergenic region</td></tr>
</table>

<table>
<tr>
<td><pre>
5922814 TCCGGCGGTCTCCGGAGAAGCCGATCCACCGGGAGCGGGACGCCGGGACGACGGGACGCC 5922873
         ||||||||||||||| | | || | | | |||||||| |||||||||
5759726 TCCGGCGGTCTCCGGCG--G--GA-C----G--A-CGGGACGC--------CGGGACGCC 5759765
</pre></td>
<td></td>
<td>intergenic region</td>
</tr>
</table>
